# A New Bromelain-Based Polyenzymatic Complex Plus N-Acetylcysteine: A Promising Approach for the Treatment of Urinary Tract Infections

**DOI:** 10.3390/ijms26104639

**Published:** 2025-05-13

**Authors:** Lucia Recinella, Morena Pinti, Silvia Di Lodovico, Andrea Brenciani, Eleonora Giovanetti, Firas Diban, Mara Di Giulio, Luigi Brunetti, Sheila Leone

**Affiliations:** 1 Department of Pharmacy, “G. d’Annunzio” University, 66100 Chieti, Italy; lucia.recinella@unich.it (L.R.); morena.pinti@phd.unich.it (M.P.); silvia.dilodovico@unich.it (S.D.L.); firas.diban@unich.it (F.D.); sheila.leone@unich.it (S.L.); 2 Department of Biomedical Sciences and Public Health, Polytechnic University of Marche, 60020 Ancona, Italy; a.brenciani@univpm.it; 3 Department of Life and Environmental Sciences, Polytechnic University of Marche, 60131 Ancona, Italy; e.giovanetti@univpm.it

**Keywords:** bromelain, N-acetylcysteine, biofilm, *Escherichia coli*, urinary infections

## Abstract

Biofilm plays a crucial role in the pathogenesis and chronicity of urinary tract infections (UTIs). The present work aimed to evaluate the anti-biofilm effects of Formulation (DIF17BRO^®^ plus NAC) in combination with ciprofloxacin (CPX) on *Escherichia coli* strains. The antimicrobial activity of ciprofloxacin was evaluated by minimum inhibitory concentration (MIC) determination, and the antibiofilm effects of ciprofloxacin alone and combined with Formulation were evaluated on *E. coli* ATCC700926, *E. coli* ATCC10536, *E. coli* PNT, and *E. coli* PCA mature biofilms in terms of CFU/mL and biomass quantifications. Moreover, the potential protective effects of Formulation plus ciprofloxacin was tested in a *Galleria mellonella* in vivo infection assay. Our results underlined the increased microbial reduction in the mature biofilm in the presence of the combination Formulation and CPX, even at a lower concentration of CPX. Formulation increased the percentage of biofilm biomass reduction, inducing a disruption of the biofilm structure itself. Our present findings confirm that MIC CPX combined with Formulation also induced an antimicrobial effect in the *G. mellonella* assay. Formulation facilitated the perturbation of the biofilm polymeric matrix, enhancing the antibiotic penetration and its antimicrobial action on bacteria, underlining Formulation’s role as an enhancer of ciprofloxacin antibacterial action.

## 1. Introduction

Urinary tract infections (UTIs) represent one of the most common bacterial infections, with significant morbidity and medical costs together with a negative impact on patient’s quality of life in terms of intimate and social relationships [1,2].

Various microbial pathogens were found to be involved in UTIs’ pathophysiology, including *Staphylococcus, Pseudomonas*, Fungi and *Enterobacteriaceae*. *Escherichia coli* (*E. coli*) is considered the most frequent microbial pathogen in UTIs, with regards to those of the lower urinary tract which are frequently involved in biofilm-related diseases [3].

Recurrent UTIs (rUTIs) are closely associated with intracellular bacterial communities (IBCs), biofilms, and antibiotic-resistant strains [4,5]. Interestingly, *E. coli* is able to enter the host cells’ cytoplasm where it can multiply to form IBCs. On the other hand, the bacteria located inside the urothelial cells can invade nearby cells inducing a new IBC formation and a natural exfoliative process, which exacerbate the symptoms [6].

Biofilm plays a crucial role in the pathogenesis, persistence, and recurrence of UTIs. It is well known that a biofilm is a community of microorganisms capable of attaching to biotic or abiotic surfaces and producing an extracellular polymeric substances matrix (EPSs), which protects bacteria from stressful conditions (pH, osmolarity, host immunity) and reduces antibiotic diffusion inside the biofilm [1,7,8,9]. Furthermore, biofilms induce protective effects in *E. coli* against antibiotic treatment and immune system activity. Antibiotic resistance in *E. coli* can be up to 1000-fold compared to planktonic bacteria [3]. 

The pharmacological treatment of UTIs includes antibiotic agents, including nitrofurantoin, trimethoprim-sulfamethoxazole, pivmecillinam, fosfomycin, β-lactams, and fluoroquinolones [10]. Several studies have confirmed the emergence of resistant *E. coli* strains, which prolong antibiotic treatments. Recently, *E. coli* resistant to at least four antibiotics has been identified in fecal isolates of up to 90% of patients treated with prophylactic low-dose antibiotics for 2 weeks [1,5,9,11]. The use of antibiotics is also associated with the development of various side effects, as well as low efficacy, a high cost, and the repetition of high doses [12].

Different natural compounds have emerged as effective agents in prevention and treatment of either acute or chronic UTIs [13].

The use of effective non-antibiotic UTI prevention strategies could reduce the risk of emergence of resistant strains and subsequent difficult-to-treat clinical infections.

The advisability of using non-antibiotic preventive treatments for rUTIs has been highlighted by recent UK, European, and US guidelines to reduce the “collateral damage” of antibiotic use by minimizing the risk of resistance development [6]. Many natural compounds show interesting antibacterial activity combined with traditional antibiotics improving their bactericidal/bacteriostatic effects [14]. Additionally, natural compounds were found able to induce a wide body of properties, including anti-adhesive activity, a reduction in bacterial motility, the inhibition of bacterial growth, the prevention of biofilm formation, the protection of host urinary cells, and the enhancement of antibacterial immunity [14].

In particular, bromelain exhibits both anti-inflammatory and antibacterial properties, due to its ability to hydrolyze some peptide bonds in the bacterial cell wall. Moreover, N-acetylcysteine (NAC), with its thiol group, can disrupt the disulfide bonds in mucus, enhancing the permeability of antibiotics within biofilms [8,15].

Recently, a novel exclusive polyenzyme complex with proteolytic activity like bromelain (DIF17BRO^®^) in combination with N-acetylcysteine (NAC) (Formulation, DIF17BRO^®^ 5 mg/mL + NAC 5 mg/mL) was found effective in inhibiting biofilm formation as well as in inducing anti-inflammatory and antioxidant activities in both in vitro and ex vivo studies [16]. In particular, DIF17BRO^®^ is a blend of fruit and stem extracts of *Ananas comosus* (L.) Merr. with an enzymatic activity of 1.825 GDU/g.

The present work aimed to evaluate the antimicrobial and anti-biofilm effects of Formulation in combination with ciprofloxacin (CPX) on various reference and clinical *E. coli* strains. In addition, we evaluated the potential protective effects of Formulation in combination with ciprofloxacin in an in vivo *Galleria mellonella* infection.

## 2. Results and Discussion

Recurrent bacterial infections associated with biofilms remain a challenging issue that is difficult to resolve. What makes biofilms difficult to treat is also the extracellular matrix component, whose composition varies depending on the microbial species and the microenvironment in which the biofilm develops, presenting a true challenge for the treatment [17]. Developing appropriate strategies to target these components is an important goal [18].

The use of natural plants as sources of bioactive compounds is a widely used method to treat microbial infections. The importance of natural compounds has increased in recent times because of the search for non-antibiotic methods to limit antibiotics use and counteract the antimicrobial resistance issue or to supplement antibiotic therapy [19].

In this study, the effects of Formulation (DIF17BRO^®^ 5 mg/mL plus NAC 5 mg/mL), combined with ciprofloxacin against mature biofilms of reference and clinical *E. coli* strains from UTIs, were assessed.

In our experimental approach, ciprofloxacin (CPX) showed Minimum Inhibitory Concentration (MIC) values of 64 mg/L for *E. coli* ATCC 700926, 3 mg/L for *E. coli* ATCC 10536, 8 mg/L for *E. coli* PNT, and 0.0078 mg/L for *E. coli* PCA.

**Figure 1:**
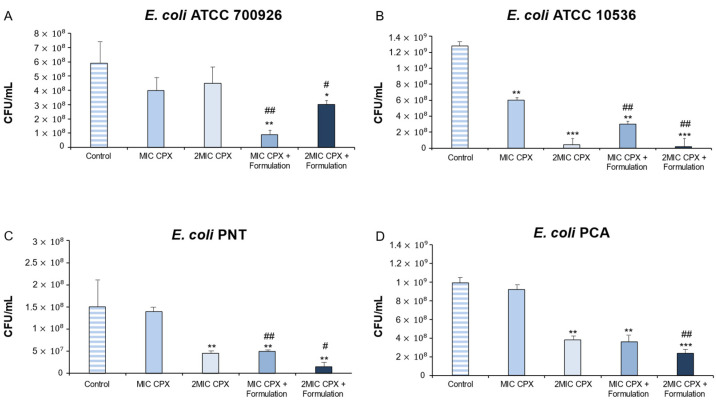
Colony forming units/mL (CFU/mL) determination of *Escherichia coli* strains (ATCC 700926, ATCC 10536, PNT, and PCA) in mature biofilm after treatment for 24 h with ciprofloxacin (MIC CPX and 2MIC CPX) and ciprofloxacin combined with Formulation (MIC CPX + Formulation and 2MIC CPX + Formulation), with respect to the untreated mature biofilm (control). * *p* < 0.05, ** *p* < 0.01, *** *p* < 0.005 vs. control; ^#^ *p* < 0.05, ^##^ *p* < 0.01 vs. MIC CPX and 2MIC CPX, respectively.

As shown in Figure 1, Formulation combined with CPX significantly reduced the CFU/mL values in all reference and clinical strains of *E. coli* as compared with CPX alone treatment.

After 24 h of treatment, ciprofloxacin alone (MIC CPX and 2MIC CPX) was not able to significantly reduce the colony forming units CFU/mL in the mature biofilm in *E. coli* ATCC 700926. On the other hand, Formulation was able to enhance CPX antimicrobial activity, resulting in a reduction in the microbial population of 83.3% and 50% (MIC CPX + Formulation, 2MIC CPX + Formulation, respectively) (Figure 1, panel A).

As regards *E. coli* ATCC 10536, the treatment with MIC CPX and 2MIC CPX resulted in a 52.1% and 97.65% of CFU/mL reduction, respectively. However, when CPX was combined with Formulation, the reduction in microbial population increased to 76.9% (for MIC CPX + Formulation) and 99.85% (for 2MIC CPX + Formulation), highlighting a possible synergistic activity between CPX and Formulation. In this context, we can suggest that Formulation induces a more effective permeation of the biofilm matrix (Figure 1, panel B).

Interestingly, the treatment for 24 h with MIC CPX alone did not significantly reduce the CFU/mL in mature biofilm for the *E. coli* PNT strain (only 8.9% of CFU/mL reduction), a multidrug-resistant strain. Intriguingly, MIC CPX combined with Formulation reduced the 70% CFU/mL, data comparable to the 72% CFU/mL reduction observed with 2MIC CPX alone. In addition, 2MIC CPX + Formulation significantly reduced the microbial population by 83.3%, compared to CPX 2MIC. Therefore, Formulation can potentiate the antibacterial CPX action, managing to achieve the same CFU/mL reduction percentage as the 2MIC CPX condition but with half of the CPX concentration (Figure 1, Panel C).

Finally, for the *E. coli* PCA strain, the presence of Formulation induced a significant increase in CFU/mL percentage reduction in the mature biofilm, confirming its ability to enhance antimicrobial action when combined with the antibiotic. In *E. coli* PCA, MIC CPX induced 9.9% of CFU/mL reduction in mature biofilm, while MIC CPX + Formulation induced a 62% CFU/mL reduction. As regards 2MIC CPX, it caused a 60% reduction in CFU/mL, while the association with Formulation resulted in a 70% reduction (Figure 1, Panel D). In our previous work [16], except for *E. coli* PNT, the treatment of *E. coli* strains’ mature biofilms with Formulation alone induced a lower or similar CFU/mL reduction for *E. coli* ATCC 700926, *E. coli* ATCC 10536, and *E. coli* PNT (13.3%, 25%, and 66.7% reduction vs. control, respectively) with respect to the condition in which Formulation was added to CPX. In the present study, results underline Formulation’s role as an enhancer of ciprofloxacin antibacterial action; in fact, when used in combination, it promoted an increased microbial reduction in the mature biofilm even at a lower concentration of CPX. Therefore, Formulation might allow us to use lower antibiotic concentrations, reducing the toxicity and negative environmental impact.

**Figure 2:**
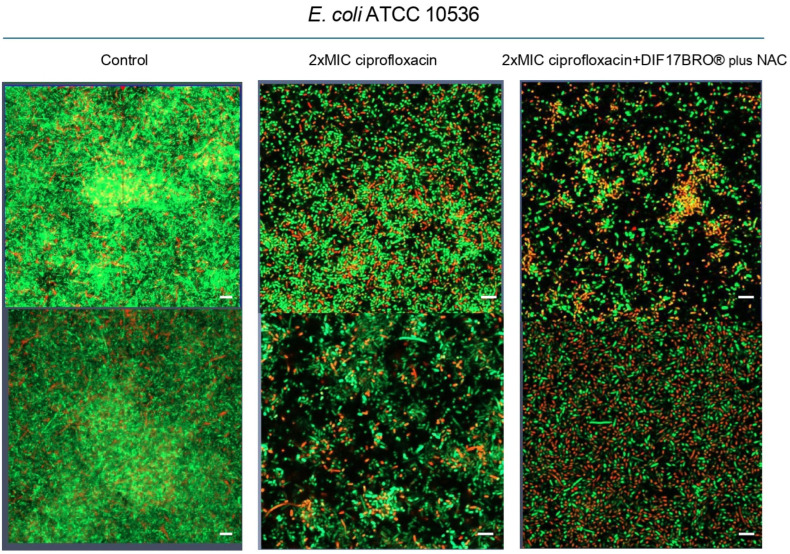
Representative images of *E. coli* ATCC 10536 control culture and that treated with 2MIC CPX alone and combined with Formulation (DIF17BRO^®^ plus NAC) obtained with a Live/Dead staining kit, confocal microscopy (600× original magnification, scale bar 10 μm).

Figure 2 shows representative images of *E. coli* ATCC 10536, the best biofilm producer, after treatment with 2MIC CPX alone and combined with Formulation. The biofilm structure in the control (untreated biofilm) appeared well-organized and structured. Confocal microscopy showed the presence of numerous bacteria alive (green) and a few dead (red) bacteria, more adherent clustered cells, and the larger aggregates displayed a high number of viable cells that seem to form a network-like structure. By contrast, after 24 h of 2MIC CPX treatment, the mature biofilm showed small aggregates with prevalent red-dead cells, tending to a disintegrated form. Interestingly, the biofilm was more disaggregated and showed a larger number of dead bacteria when treated with 2MIC CPX + Formulation, indicating that Formulation seems to potentiate the antibacterial/anti-biofilm action of ciprofloxacin.

In the present study, we also evaluated the ability of ciprofloxacin (MIC CPX and 2MIC CPX) alone and combined with Formulation to perform a disruptive/permeating activity on the mature biofilm. The biomass reduction data reflect the results of the antimicrobial activity on the *E. coli* mature biofilm.

**Figure 3:**
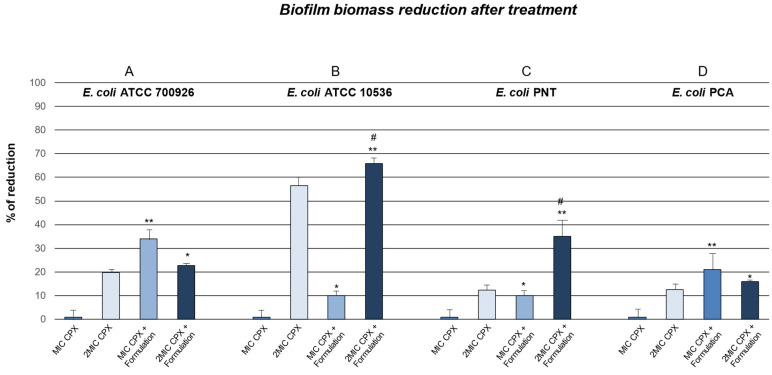
Percentage of reduction in biofilm biomass of mature biofilm after treatment for 24 h with ciprofloxacin (MIC CPX and 2MIC CPX) and ciprofloxacin combined with Formulation (MIC CPX + Formulation and 2MIC CPX + Formulation), with respect to untreated mature biofilm (control). * *p* < 0.05, ** *p* < 0.01, vs. control; ^#^ *p* < 0.05 vs. 2MIC CPX.

As shown in Figure 3, for all strains tested, Formulation increased the percentage of biofilm biomass reduction, inducing a disruption of the biofilm structure itself. The percentage of biofilm biomass reduction was calculated compared to the control (untreated mature biofilm).

As reported in our previous work [16], in which Formulation was tested alone, Formulation facilitated the perturbation of the biofilm polymeric matrix, enhancing the antibiotic penetration and thus its antimicrobial action on bacteria (Figure 3).

In particular, after 24 h of treatment, ciprofloxacin alone significantly increased biomass biofilm reduction percentage in *E. coli* ATCC 700926 with respect to untreated mature biofilm but only at higher concentrations (2MIC CPX). On the other hand, Formulation was able to enhance biomass biofilm reduction percentage induced by CPX at both concentrations (Figure 3, panel A).

Similarly, in *E. coli* ATCC 10536, when CPX was combined with Formulation, the biomass biofilm reduction percentage significantly increased with respect to treatment with CPX alone at both concentrations (Figure 3, panel B).

The effects of CPX combined with Formulation in biomass biofilm reduction percentage were also investigated in clinical *E. coli* multidrug resistant PNT and PCA strains.

Intriguingly, the treatment with MIC CPX alone only induced a 1% biomass biofilm reduction percentage in clinical PNT and PCA strains (Figure 3, panels C and D). On the other hand, CPX at higher concentrations (CPX 2MIC) significantly increased the biomass biofilm reduction percentage in the same clinical *E. coli* strains (Figure 3, panels C and D). When CPX (MIC and 2MIC) was used in combination with Formulation, we showed a significant increase in biomass biofilm reduction percentage compared to CPX in both PNT and PCA strains (Figure 3, panels C and D).

In a recent study, Zhang et al. (2024) [20] underlined the intricate complex super-structure of the uropathogenic *Escherichia coli* (UPEC) biofilm. In the urinary tract, biofilms exhibit low wettability, resulting from the synergistical combination among cells, and curli structures formed on cellulose scaffolds that lead to enhanced surface hydrophobicity. In this way, resistance to wetting may prevent and hinder the host-derived antimicrobial agents and antibiotics limiting access to the inner part of the microbial biofilm. Additionally, partially excluding biofilm from the aqueous environment could help prevent the detachment of biofilms due to urine flow and the access to water-soluble antimicrobial agents [20].

As known, the extracellular matrix of the *E. coli* biofilm is an intricate network composed of polysaccharides, proteins, and extracellular DNA, which work synergistically to provide structural stability, protecting bacteria from environmental stresses, and contribute to antibiotic resistance/tolerance. In particular, the *E. coli* biofilm matrix, similarly to *Salmonella*, is characterized by the presence of fibril-forming amyloid protein curli and cellulose [21,22].

Our results emphasize Formulation’s capability to interact with the antibiotic action by implementing its combined action against the mature biofilm attributable to the presence of bromelain, for its proteolytic action, and that of NAC, which exhibits mucolytic activity. Bromelain, the cysteine protease from pineapple (*Ananas comosus*) fruit and stem, contains proteases, esterase, cellulases, and peroxidases [23], has broad therapeutic use, and is able to hydrolyze substrates like fibrin, collagen, casein, and elastin. Its antimicrobial activity could be correlated to its hydrolyzation of some peptide bonds in the bacterial cell wall, allowing the cell to escape from the wall, swell with water, and open [24]. Moreover, bromelain is able to inhibit the enterotoxin production of *E. coli* [15], and it was shown that the combined use of bromelain and antibiotics increased the antibacterial effect due to the grater absorption of antibiotics induced by bromelain, leading to improved drug distribution in the microbial cells [15].

N-acetylcysteine (NAC) is an amino-thiol with antioxidant and mucolytic activity [25]. El-Feky et al. showed the NAC ability to increase the therapeutical activity of ciprofloxacin by degrading the extracellular polysaccharide matrix of biofilms of *Staphylococcus aureus*, *S. epidermidis*, *E. coli*, *Klebsiella pneumoniae*, *Pseudomonas aeruginosa*, and *Proteus vulgaris*, isolated from a ureteral stent [24,26].

The combination of bromelain with NAC has already been shown to act as effective antibiofilm agents on *P. aeruginosa* [25].

In the second experimental step, we evaluated the capacity of ciprofloxacin (CPX) alone and combined with Formulation (CPX + Formulation) to counteract the *E. coli* infection in a *Galleria mellonella* larva in vivo model.

The *Galleria mellonella* model is a recognized model to study *E. coli* infection due to these larvae’s innate immune responses and physiological similarities to higher organisms [27]. Moreover, studies have demonstrated that *G. mellonella* larvae are susceptible to *E. coli* infections, making them a valuable tool for investigating bacterial virulence and testing antimicrobial agents [28].

**Figure 4:**
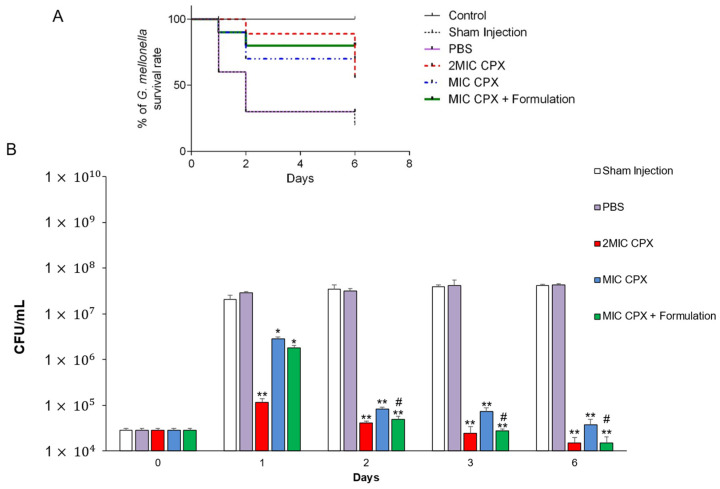
In vivo infection assay in *Galleria mellonella* larvae. (panel **A**) Kaplan–Meyer survival curves of *G. mellonella* larva infected with *E. coli* PNT clinical strain for 1 h, then treated with PBS, ciprofloxacin (MIC CPX and 2MIC CPX) and MIC CPX combined with Formulation that were checked every day until 6 days. (panel **B**) Recovery of *E. coli* clinical strain CFU/larva in *G. mellonella* at different time points and different conditions. * *p* < 0.05, ** *p* < 0.01 vs. vehicle; ^#^ *p* < 0.05 vs. MIC CPX.

Figure 4 shows *G. mellonella* survival rate infected with *E. coli* PNT and treated with CPX alone (MIC and 2MIC) and combined with Formulation.

Treatment with 2MIC CPX and MIC CPX showed a protective effect against *E. coli* infection with a *G. mellonella* survival rate of up to 80% and 60% after 6 days, respectively (Figure 4, panel A). In particular, 2MIC CPX combined with Formulation showed a stable effect in time, protecting *G. mellonella* from *E. coli* infection with a percentage of larvae survival of 70% (6 days). The differences were compared with Log-rank test and the survival curves were statistically significant. These results were confirmed by the *E. coli* PNT CFU/mL recovered from *G. mellonella* at different times. As shown in Figure 4, panel B, a statistically significant *E. coli* PNT CFU/mL reduction was obtained following treatment with 2 MIC CPX, MIC CPX and MIC CPX + Formulation for 1 day with respect to the vehicle at each contact time. The treatment with MIC CPX combined with Formulation displayed a remarkable effect in terms of *E. coli* PNT CFU/mL reduction starting from day 2 with respect to MIC CPX alone. These findings confirmed that MIC CPX combined with Formulation also induced an antimicrobial effect in vivo. In particular, this combination was found as effective as 2MIC CPX in reducing *E. coli* PNT CFU/mL recovered from *G. mellonella*.

On the basis of these results, the beneficial effects of the pretreatment with our Formulation was tested in clinical multidrug-resistant *E. coli* PNT strain.

This last treatment was included in the study to further evaluate the ability of Formulation to facilitate the antibiotic permeation through the biofilm matrix.

**Figure 5:**
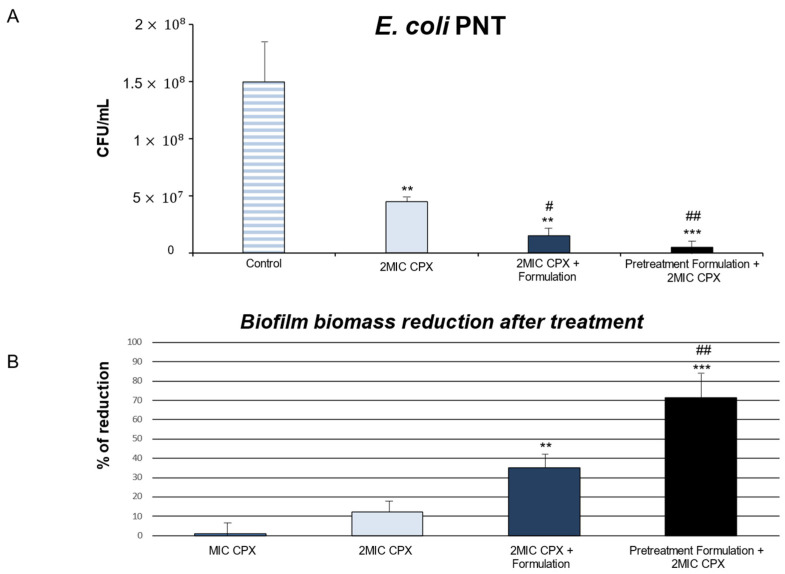
Colony forming units/mL (CFU/mL) determination of *Escherichia coli* PNT strain in mature biofilm (panel **A**) and percentage of reduction in biofilm biomass after treatment for 24 h (panel **B**) with ciprofloxacin (MIC CPX and 2MIC CPX) and ciprofloxacin combined with Formulation (2MIC CPX + Formulation) with respect to untreated mature biofilm (Control). Pretreatment condition for 5 h and then addition of 2xMIC ciprofloxacin (pretreatment Formulation + 2MIC CPX). ** *p* < 0.01, *** *p* < 0.005 vs. control; ^#^ *p* < 0.05, ^##^ *p* < 0.01 vs. 2MIC CPX.

Therefore, the mature biofilm was treated with Formulation for 5 h and then 2MIC ciprofloxacin was added (pretreatment Formulation + 2MIC CPX). For the *E. coli* PNT strain, it was particularly interesting to note how pretreatment with Formulation maximized the overall antimicrobial action in the mature biofilm, underlining its capability to facilitate the permeation of antibiotics (94.3% reduction with respect to the control) (Figure 5, panel A). As regards biofilm biomass reduction, following the 5 h pretreatment with Formulation, an interesting penetrating and matrix-disrupting capacity was observed with a percentage of biomass reduction of 70% for *E. coli* PNT compared to the condition in which Formulation was used with an antibiotic at the same time. Formulation pretreatment could represent a good matrix destabilizer, increasing the susceptibility of biofilms to ciprofloxacin.

Our results emphasize Formulation’s role as enhancer of the ciprofloxacin antibacterial role; in fact, when used in combination with CPX, Formulation promoted an increased microbial reduction in the mature biofilm even at a low concentration of MIC ciprofloxacin. This combination could allow us to use a lower antibiotic amount, reducing the ciprofloxacin toxicity.

## 3. Materials and Methods

### 3.1. Bacterial Cultures

The bacterial strains used in this study consisted of two reference strains, *E. coli* ATCC 700926 and *E. coli* ATCC 10536, and two clinical isolates, *E. coli* PNT and *E. coli* PCA, obtained from the private collection of Bacteriological Laboratories of the Pharmacy Department, “G. d’Annunzio” University Chieti-Pescara. The clinical strains were obtained from urine cultures of patients with recurrent urinary tract infections. The strains were isolated and identified using the Vitek2 system

Bacteria were grown on MacConkey agar (MK, Oxoid, Milan, Italy) and fresh, pure bacterial colonies were cultured in Trypticase Soy Broth (TSB, Oxoid, Milan, Italy) and incubated aerobically at 37 °C overnight. For the antimicrobial test, the overnight cultures were refreshed in Mueller–Hinton Broth cation-adjusted 2 (MHB, Oxoid, Milan, Italy) for 2 h at 37 °C in an orbital shaker in aerobic condition and standardized at ~5 × 10^7^ CFU/mL. For the anti-biofilm test, the overnight cultures were refreshed in TSB + 1% glucose and standardized to OD_600_ 0.125.

The standardized broth cultures were used in the experiments.

### 3.2. Antimicrobial Activity of Ciprofloxacin

The antibacterial effect of ciprofloxacin was assessed by Minimum Inhibitory Concentration (MIC) and Minimum Bactericidal Concentration (MBC) determinations according to CLSI guidelines [29]. Ciprofloxacin (FLUKA-Biochemika, Buchs, Switzerland) was diluted to obtain the final concentration ranging from 128 to 0.0039 mg/L. The plates were incubated in aerobic conditions for 24 h. The MIC was defined as the lowest concentration of ciprofloxacin that completely inhibited growth while the MBC was determined as the lowest concentration that inhibited bacterial growth after cultivation on Mueller–Hinton Agar plates (MHA, Oxoid, Milan, Italy).

### 3.3. Anti-Biofilm Effect of Ciprofloxacin Alone and in Combination with Formulation (DIF17BRO^®^ Plus NAC)

The antibiofilm effect of ciprofloxacin alone and combined with Formulation (DIF17BRO^®^ plus NAC) [16] was evaluated on *E. coli* ATCC 700926, *E. coli* ATCC 10536, *E. coli* PNT, and *E. coli* PCA mature biofilms. Briefly, 200 μL of standardized broth cultures in TSB plus 1% glucose was inoculated in a 96-well flat-bottomed polystyrene microtiter plate under aerobic conditions for 24 h. After incubation, the supernatants were removed, and the biofilm were washed with PBS (Merk, KGaA, Darmstadt, Germany) and treated with ciprofloxacin (CPX) at MIC and 2MIC concentrations (MIC CPX and 2MIC CPX) alone and combined with Formulation (DIF17BRO^®^ 5 mg/mL + NAC 5 mg/mL), (MIC CPX + Formulation and 2MIC CPX + Formulation). Finally, the antibiofilm effect of the pretreatment with the Formulation condition was evaluated in the *E. coli* PNT strain. The mature biofilm was treated with Formulation for 5 h, and then ciprofloxacin 2MIC was added (pretreatment Formulation + 2MIC CPX). The plates were incubated for 24 h at 37 °C, and after incubation, the adherent CFU/mL and biomass were determined. For the CFU/mL, each well was scraped, and the adherent bacteria were diluted and spread on MK. For the biomass quantification, the dried biofilms were stained for 1 min with 0.1% Safranin, eluted with ethanol, and the biomass was measured by ELISA reader OD_492_ nm.

All experiments were performed three times independently.

The treated and untreated mature biofilms were also observed by using Confocal Laser Scanning Microscopy (CLSM, Zeiss LSM510 META confocal system (Jena, Germany)) with Live/Dead staining kit according to Di Fermo et al. (2021) [30].

For the CLSM analysis, 2000 μL of standardized broth cultures in TSB plus 1% glucose was inoculated in a 3.5 cm diameter flat-bottomed polystyrene well plate and incubated under aerobic conditions for 24 h. After incubation, the supernatants were removed and the biofilm were washed with PBS (Merk, KGaA, Darmstadt, Germany) and treated with ciprofloxacin (CPX) at MIC and 2MIC concentrations (MIC CPX and 2MIC CPX) alone and combined with Formulation (DIF17BRO^®^ 5 mg/mL + NAC 5 mg/mL), (MIC CPX + Formulation and 2MIC CPX + Formulation). Finally, the antibiofilm effect of the pretreatment with the Formulation condition was evaluated in the *E. coli* PNT strain. The mature biofilm was treated with Formulation for 5 h, and then ciprofloxacin 2MIC was added (pretreatment Formulation + 2MIC CPX). The plates were incubated for 24 h at 37 °C, and after incubation, the supernatant was removed, and the adherent bacteria were stained with the Live/Dead kit. The samples were examined by a Zeiss LSM510 META confocal system (Jena, Germany) connected to an inverted Zeiss Axiovert 200 microscope equipped with Plan Neofluar oil-immersion objectives (63⁄1Æ4 and 100⁄1Æ45 NA). The green fluorescence of SYTO 9 and red fluorescence of Propidium iodide (PI) were excited using an argon laser beam with excitation lines at 488 nm (6% of potency) and a helium⁄neon 543 nm source (10% of potency). To separate the emissions of fluorochromes, HTF 488⁄543 and NTF 545, primary and secondary dichroic mirrors were used. Detector band-pass filters were set over 505–530 and 565–615 ranges for the green and red emissions that were alternatively recorded using the MultiTrack acquisition to avoid overlapping in the fluorochromes emission. All experiments were performed at room temperature.

### 3.4. In Vivo Galleria Mellonella Infection Assay

To evaluate the potential protective effect of ciprofloxacin alone and combined with Formulation (DIF17BRO^®^ plus NAC) against *E. coli* infection, the in vivo *Galleria mellonella* infection assay was performed according to Di Lodovico et al. (2019) [31], with some modifications.

Briefly, seven groups of ten randomly selected *G. mellonella* larvae were injected with 10 μL of *E. coli* PNT (~10^6^ CFU/mL) in the last left proleg, and after 1 h, the larvae were treated in the last right proleg as follows: ten larvae with sham injection; ten larvae with 10 μL of PBS; ten with 10 μL of MIC and 2MIC ciprofloxacin (MIC CPX and 2MIC CPX, respectively); ten larvae with 10 μL of CPX combined with 10 μL of Formulation (MIC CPX + Formulation). Ten uninjected larvae were used as the control group. Larvae were incubated at 37 °C in the dark, and the *G. mellonella* survival rate were checked every day until day 6. The larvae did not receive nutrition, and the unresponsive black larvae were considered dead. The *E. coli* capability to infect *G. mellonella* in the presence of all the above conditions was also evaluated by CFU/mL determination. Every day, one larva of each condition was chilled on ice for 5 min, washed, its haemocoel was serially diluted, and the CFUs was determined by spreading on MK. The plates were incubated at 37 °C in an aerobic environment.

### 3.5. Statistical Analysis

Data were obtained from independent experiments performed in triplicate. Data are shown as the means ± standard deviation (SD). Differences between groups were assessed with a one-way analysis of variance (ANOVA) (*p* values ≤ 0.05 were considered statistically significant) followed by the Bonferroni post hoc test. Survival curves were plotted using the Kaplan–Meier method, and survival differences were calculated using the Log-rank test for multiple comparisons. GraphPad Prism version 9.0 (Graph-pad Software Inc., San Diego, CA, USA) was used to fit a curve to the infection data.

## 4. Conclusions

The growing emergence of antibiotic-resistant and tolerant bacteria poses a serious challenge to public health, requiring both responsible antibiotic management and the development of innovative therapeutic approaches. Many natural compounds showed interesting antibacterial activity also combined with traditional antibiotics, improving their bactericidal/bacteriostatic effects [14,32].

Based on the interesting antibiofilm and antibacterial actions, despite the limitation of this study related to the low number of clinical *E. coli* strains, it can be concluded that Formulation can be considered an interesting candidate for effective therapeutic schemes for the treatment of urinary tract infections. Moreover, it is important to focus on the eco-sustainability of Formulation, which is produced by taking advantage of plant extract coming from different parts of the plant following green methods. As reported by Sha et al., among food waste, fruit waste represents a current perspective on the sustainable generation of pharmacological, nutraceutical, and bioactive compounds; for instance, around 40% of pineapple waste is represented by pineapple peel, rich in epicatechins, ferulic acids, gallic acid, and active anti-oxidant compounds representing a source of prebiotic elements to boost the growth of healthy intestinal, vaginal, and urinary tract microbiota [33].

Our results demonstrated that Formulation combined with ciprofloxacin promoted an increased microbial reduction in the mature biofilm compared to ciprofloxacin alone. Moreover, this combination, destabilizing the biofilm matrix, enhanced the antibacterial activity of the antibiotic in the bacterial sessile phase. This allowed us to use a lower antibiotic dose, reducing both the potential toxicity induced by ciprofloxacin as well as antibiotic resistance.

## Data Availability

The original contributions presented in this study are included in the article. Further inquiries can be directed to the corresponding author(s).

## References

[1] Mancuso G., Midiri A., Gerace E., Marra M., Zummo S., Biondo C. (2023). Urinary Tract Infections: The Current Scenario and Future Prospects. Pathogens.

[2] Lila A.S.A., Rajab A.A.H., Abdallah M.H., Rizvi S.M.D., Moin A., Khafagy E.-S., Tabrez S., Hegazy W.A.H. (2023). Biofilm Lifestyle in Recurrent Urinary Tract Infections. Life.

[3] Ballén V., Cepas V., Ratia C., Gabasa Y., Soto S.M. (2022). Clinical *Escherichia coli*: From Biofilm Formation to New Antibiofilm Strategies. Microorganisms.

[4] Kim A., Ahn J., Choi W.S., Park H.K., Kim S., Paick S.H., Kim H.G. (2021). What is the Cause of Recurrent Urinary Tract Infection? Contemporary Microscopic Concepts of Pathophysiology. Int. Neurourol. J..

[5] Sheikhi R., Amini M.E., Alidoust L., Atrkar Roushan Z., Nikokar I. (2024). Evaluation of adhesin genes and risk factors associated with urinary tract infections by drug-resistant uropathogenic *Escherichia coli* in North of Iran. J. Infect. Dev. Ctries..

[6] Robino L., Sauto R., Morales C., Navarro N., González M.J., Cruz E., Neffa F., Zeballos J., Scavone P. (2024). Presence of intracellular bacterial communities in uroepithelial cells, a potential reservoir in symptomatic and non-symptomatic people. BMC Infect. Dis..

[7] Alshaikh S.A., El-Banna T., Sonbol F., Farghali M.H. (2024). Correlation between antimicrobial resistance, biofilm formation, and virulence determinants in uropathogenic *Escherichia coli* from Egyptian hospital. Ann. Clin. Microbiol. Antimicrob..

[8] Amante C., De Soricellis C., Luccheo G., Luccheo L., Russo P., Aquino R.P., Del Gaudio P. (2023). Flogomicina: A Natural Antioxidant Mixture as an Alternative Strategy to Reduce Biofilm Formation. Life.

[9] Mancini A., Pucciarelli S., Lombardi F.E., Barocci S., Pauri P., Lodolini S. (2020). Differences between Community—And Hospital—Acquired urinary tract infections in a tertiary care hospital. New Microbiol..

[10] Jancel T., Dudas V. (2002). Management of Uncomplicated Urinary Tract Infections. West. J. Med..

[11] Olin S.J., Bartges J.W. (2015). Urinary tract infections: Treatment/comparative therapeutics. Vet. Clin. N. Am. Small Anim. Pract..

[12] Shaheen G., Akram M., Jabeen F., Ali Shah S.M., Munir N., Daniyal M., Riaz M., Tahir I.M., Ghauri A.O., Sultana S. (2019). Therapeutic Potential of Medicinal Plants for the Management of Urinary Tract Infection: A Systematic Review. Clin. Exp. Pharmacol. Physiol..

[13] Das S. (2020). Natural Therapeutics for Urinary Tract Infections-a Review. Future J. Pharm. Sci..

[14] Zhou Y., Zhou Z., Zheng L., Gong Z., Li Y., Jin Y., Huang Y., Chi M. (2023). Urinary Tract Infections Caused by Uropathogenic *Escherichia coli*: Mechanisms of Infection and Treatment Options. Int. J. Mol. Sci..

[15] Chakraborty A.J., Mitra S., Tallei T.E., Tareq A.M., Nainu F., Cicia D., Dhama K., Emran T.B., Simal-Gandara J., Capasso R. (2021). Bromelain a Potential Bioactive Compound: A Comprehensive Overview from a Pharmacological Perspective. Life.

[16] Recinella L., Pinti M., Libero M.L., Di Lodovico S., Veschi S., Piro A., Generali D., Acquaviva A., Nilofar N., Orlando G. (2024). Beneficial Effects Induced by a Proprietary Blend of a New Bromelain-Based Polyenzymatic Complex Plus N-Acetylcysteine in Urinary Tract Infections: Results from In Vitro and Ex Vivo Studies. Antibiotics.

[17] Pecoraro C., Carbone D., Parrino B., Cascioferro S., Diana P. (2023). Recent Developments in the Inhibition of Bacterial Adhesion as Promising Anti-Virulence Strategy. Int. J. Mol. Sci..

[18] Flemming H.-C., van Hullebusch E.D., Neu T.R., Nielsen P.H., Seviour T., Stoodley P., Wingender J., Wuertz S. (2023). The biofilm matrix: Multitasking in a shared space. Nat. Rev. Microbiol..

[19] Vaou N., Stavropoulou E., Voidarou C., Tsigalou C., Bezirtzoglou E. (2021). Towards Advances in Medicinal Plant Antimicrobial Activity: A Review Study on Challenges and Future Perspectives. Microorganisms.

[20] Zhang T., Ray S., Melican K., Richter-Dahlfors A. (2024). The maturation of native uropathogenic *Escherichia coli* biofilms seen through a non-interventional lens. Biofilm.

[21] Römling U. (2005). Characterization of the rdar morphotype, a multicellular behaviour in Enterobacteriaceae. Cell. Mol. Life Sci..

[22] Serra D.O., Richter A.M., Hengge R. (2013). Cellulose as an architectural element in spatially structured *Escherichia coli* biofilms. J. Bacteriol..

[23] Wang S.L., Lin H.T., Liang T.W., Chen Y.J., Yen Y.H., Guo S.P. (2008). Reclamation of chitinous materials by bromelain for the preparation of antitumor and antifungal materials. Bioresour. Technol..

[24] Jančič U., Gorgieva S. (2021). Bromelain and Nisin: The Natural Antimicrobials with High Potential in Biomedicine. Pharmaceutics.

[25] Carter C.J., Pillai K., Badar S., Mekkawy A.H., Akhter J., Jefferies T., Valle S.J., Morris D.L. (2021). Dissolution of Biofilm Secreted by Three Different Strains of *Pseudomonas aeruginosa* with Bromelain, *N*-Acetylcysteine, and Their Combinations. Appl. Sci..

[26] El-Feky M.A., El-Rehewy M.S., Hassan M.A., Abolella H.A., Abd El-Baky R.M., Gad G.F. (2009). Effect of ciprofloxacin and N-acetylcysteine on bacterial adherence and biofilm formation on ureteral stent surfaces. Pol. J. Microbiol..

[27] Tsai C.J.-Y., Loh J.M.S., Proft T. (2016). *Galleria mellonella* infection models for the study of bacterial diseases and for antimicrobial drug testing. Virulence.

[28] Sugeçti S. (2021). Biochemical and immune responses of model organism *Galleria mellonella* after infection with *Escherichia coli*. Entomol. Exp. Appl..

[29] Clinical and Laboratory Standards Institute [CLSI] (2018). Methods for Dilution Antimicrobial Susceptibility Tests for Bacteria That Grow Aerobically.

[30] Di Fermo P., Ciociola T., Di Lodovico S., D’ercole S., Petrini M., Giovati L., Conti S., Di Giulio M., Cellini L. (2021). Antimicrobial Peptide L18R Displays a Modulating Action against Inter-Kingdom Biofilms in the Lubbock Chronic Wound Biofilm Model. Microorganisms.

[31] Di Lodovico S., Napoli E., Di Campli E., Di Fermo P., Gentile D., Ruberto G., Nostro A., Marini E., Cellini L., Di Giulio M. (2019). *Pistacia vera* L. oleoresin and levofloxacin is a synergistic combination against resistant *Helicobacter pylori* strains. Sci. Rep..

[32] Romano L., Napolitano L., Crocetto F., Sciorio C., Priadko K., Fonticelli M., Federico A., Romano M., Gravina A.G. (2024). The potential therapeutic role of Hericium erinaceus extract in pathologic conditions involving the urogenital-gut axis: Insights into the involved mechanisms and mediators. J. Physiol. Pharmacol..

[33] Sha S.P., Modak D., Sarkar S., Roy S.K., Sah S.P., Ghatani K., Bhattacharjee S. (2023). Fruit waste: A current perspective for the sustainable production of pharmacological, nutraceutical, and bioactive resources. Front. Microbiol..

